# Evidence for Glass and Spin-Glass Phase Transitions From the Dynamic Susceptibility

**DOI:** 10.6028/jres.102.016

**Published:** 1997

**Authors:** D. Bitko, S. N. Coppersmith, R. L. Leheny, N. Menon, S. R. Nagel, T. F. Rosenbaum

**Affiliations:** The James Franck Institute and Department of Physics, The University of Chicago, Chicago, IL 60637

**Keywords:** dielectric susceptibility, glass transition, magnetic susceptibility, nonlinear susceptibility, specific-heat spectroscopy, spin-glass transition

## Abstract

We present evidence that there is a phase transition, with a diverging static susceptibility, underlying the transformation of a liquid into a glass. The dielectric susceptibility, at frequencies above its characteristic value, shows a power-law tail extending over many decades to higher frequencies. An extrapolation of this behavior to the temperature where the dynamics becomes arrested indicates a diverging susceptibility. We present evidence for analogous behavior in the magnetic susceptibility of a paramagnet approaching the spin-glass transition. The similarity of the response in these two glassy systems suggests that some conventional lore, such as that the spin glass shows evidence for a diverging correlation length only in a nonlinear but not in the linear susceptibility, may be invalid.

## 1. Introduction^3^

A classical liquid supercooled far enough below its freezing temperature will have immeasurably slow dynamics [1]. The transition between the liquid and this glassy state is not well understood. For example, the cause of the dramatic growth with decreasing temperature of the viscosity and its associated time scales and whether these quantities actually diverge at a finite temperature are issues that are still hotly debated. Unfortunately, a question such as whether there is a true divergence in the relaxation cannot be answered directly because at temperatures far (tens of degrees Kelvin) above the point, *T*_0_, where such a divergence appears plausible in the data, the relaxation times become so enormous as to make thermodynamic equilibrium experimentally inaccessible. The question as to whether there is a phase transition that can be associated with an infinitely slow cooling of the system, therefore, can only be addressed by looking at the subtle effects in the data at higher temperature which then need to be extrapolated to lower temperatures near *T*_0_.

In spin glasses, it is possible to get much closer to the transition temperature, *T*_sg_, than is possible for structural glasses [2]. For these systems there is considerable evidence, both experimental and theoretical, that a true underlying phase transition exists [3]. An experimental manifestation of this transition is the divergence at *T*_sg_ of the static nonlinear magnetic susceptibility. The linear susceptibility, by contrast, has a cusp near *T*_sg_ and does not appear to grow rapidly with decreasing temperature. Thus it has been assumed that, although the nonlinear susceptibility diverges, the linear susceptibility does not.

Extensive measurements of the dielectric susceptibility [4] and specific heat [5, 6], of supercooled liquids provide insight as to what is happening as the times scales increase on lowering the temperature [7]. In particular, the dielectric measurements cover a wide range of frequency so that they can determine the shape of the response as the temperature is varied. Although much more is known about the spin-glass transition than about its structural glass counterpart, there is considerably less susceptibility data that span a very wide frequency range and none come close to covering the 14 decades of frequency used in studying supercooled liquids. This is partly due to the fact that, since many spin glasses are metallic, high-frequency data are difficult to obtain without significant eddy-current heating. *T*o remedy this situation, the magnetic susceptibility of an insulating Ising spin glass recently has been measured over a relatively wide frequency range [8] and can be compared to the behavior of the susceptibility in liquids. This data shows a number of surprising similarities between supercooled liquids approaching the glass and a paramagnet approaching the spin glass.

As indicated above, the conclusions we obtain about the glass transition must rely on extrapolations of data over a wide temperature range. The conclusions that we can reach about the spin-glass transition are not as dependent on extrapolations over wide ranges of temperature but are, on the other hand, dependent on extrapolations over a wide range of frequency. The observed similarities between these two systems give us added confidence about the procedures we have used to extrapolate our results to the region near the transition since different types of extrapolations are involved in the two cases.

The susceptibility measurements allow us to draw several perhaps surprising conclusions: 1) From the shape of the susceptibility in both the supercooled liquids and spin glasses, we conclude that the high-frequency response contains information about the approach to the transition. 2) From the form of the frequency response above the characteristic loss peak at *v*_P_ of the dielectric susceptibility in supercooled liquids we argue for the existence of a diverging static susceptibility, Δ*ε* [9]. 3) From the form of both the low- and high-frequency response of a spin glass [8], we argue that there is a divergence of the linear, as well as of the nonlinear, magnetic susceptibility at the spin-glass transition. Evidence compiled from the literature support this claim. We will outline these conclusions below.

## 2. Shape of the Dielectric Susceptibility in Liquids

**Fig. 1:**
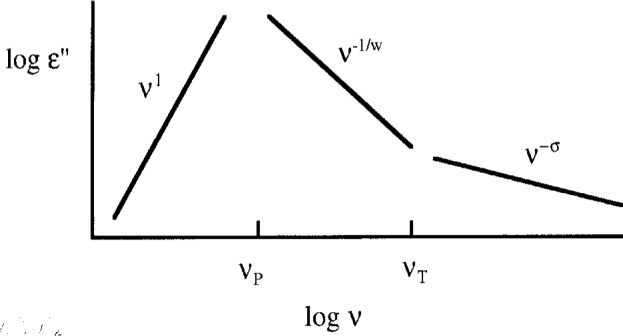
A schematic diagram showing the three distinct frequency regimes for the imaginary part of the dielectric response of a supercooled liquid.

The dielectric susceptibility data, *ε* (*v*) = *ε*′(*v*) + *iε*″(*v*), for many different supercooled liquids have shown that there are generically three distinct frequency regimes for the primary response at every temperature. Figure 1 shows a schematic version of this behavior for the imaginary part, *ε*″(*v*). At low frequency, *ε*″(*v*) increases linearly with increasing frequency before reaching a maximum in the vicinity of a characteristic frequency *v*_P_. (There has been some controversy in the literature about whether this region of the spectrum is always linear in the frequency [10]. For the argument that follows, the exponent in this region is not important.) Between *v*_P_ and a higher frequency, *v*_T_, the response decreases as *C*_1_*v*^−1/^*^w^*, where *w* is approximately the width of the peak, *W*, normalized by the width of a simple Debye relaxation, *W*_D_: *w* ≅ *W*/*W*_D_. Above *v*_T_, the response changes to a second power law, *C*_2_*v*^−^*^σ^*, with *σ* smaller than 1/*w*. Recent measurements have shown that this power law persists over at least eight decades in frequency [11]. Hence, we feel justified in regarding it as the asymptotic form for the primary response, *ε*″(*v*), that extends from the frequency *v*_T_ up to an approximately temperature-independent phonon cut-off frequency, *v*_0_, in the infrared.

## 3. Diverging Static Susceptibility in Supercooled Liquids

As the temperature is lowered, the entire spectrum shifts to lower frequency. (If a divergence in the times scales truly exists, then at *T*_0_, the entire curve will have shifted infinitely far to the left on the log *v* axis.) As the curve shifts to lower frequencies, its shape also changes: both 1/*w* and *σ* decrease [12]. An extrapolation of the data for *σ* indicates that it approaches zero (i.e., *ε*″(*v*) = *C*_2_ becomes independent of frequency) as *T* approaches *T_σ_* [9, 11]. The simplest extrapolation which is consistent with the data is that *σ* approaches zero linearly with decreasing temperature: *σ* = *D*(*T* − *T_σ_*). The extrapolations that we have done strongly suggest that *T_σ_* is close to (or slightly greater than) *T*_0_ [9, 11]. Thus we have the situation that, at *T*_0_, *ε*″(*v*) is constant over the entire range *v*_T_(= 0) ≤ *v* ≤ *v*_0_.

We can argue that the above data lead to a diverging static susceptibility, Δ*ε*, for the supercooled liquid at the transition temperature *T*_0_ = *T_σ_* [9]. This can be seen by examining the Kramers-Kronig relation:

$$\Delta \varepsilon =\frac{2}{\pi }\int_{0}^{\infty }\frac{\varepsilon^{\prime \prime }(\nu )}{\nu }d\nu =\frac{2}{\pi }\int_{-\infty }^{\infty }\varepsilon^{\prime \prime }(\nu )\;d\;\ln\;\nu .$$ (1)

We can isolate the contribution, δΔ*ε*, to the entire static susceptibility from the high-frequency tail:

δΔε=2π∫lnνTlnν0C2ν−σdlnν. (2)

If we use the common Vogel-Fulcher fitting form for the characteristic frequency *v*_T_: *v*_T_ = *v*_0_ exp(−A/(*T* − *T*_0_)), (measurements show that *v*_T_ tracks *v*_P_) and assume, as suggested above, that *σ* = *D*(*T* − *T*_0_), we then find that the static susceptibility diverges with Curie-Weiss behavior:

$$\delta \Delta \varepsilon \propto {\left(T-T_{0}\right)}^{-1}.$$ (3)

Such a result is suggestive of mean-field behavior which would be appropriate for temperatures far from the critical point. The assumption of Vogel-Fulcher behavior for the temperature dependence of *v*_T_ is not essential for this result; a power-law divergence would also lead to Curie-Weiss behavior. If *σ* were to decrease to zero with a different power of the temperature, then the divergence of Δ*ε* would have a different exponent. One final remark is that the argument for a diverging susceptibility depends on having the prefactor of the power law, *C*_2_, not go to zero at the same time as does the exponent *σ*. We also have strong evidence that this does not happen and that *C*_2_ remains finite in any reasonable extrapolation down to temperatures close to the transition [9, 11].

The Kauzmann temperature [13], where the excess entropy of the liquid over that of the crystal extrapolates to zero, has also been found to be close to the temperature where the dynamics appears to become arrested [1, 7]. It is interesting to note that the above argument thus suggests that three independently determined temperatures, each of which would suggest the existence of a phase transition, [the temperature where the high-frequency response becomes flat (at *T_σ_*), the temperature where the dynamics appears to freeze (at *T*_0_), and the Kauzmann temperature where the excess entropy vanishes (at *T*_K_)] are all coincident:

$$T_{0}≅T_{\sigma }≅T_{K}.$$ (4)

## 4. Frequency and Temperature Dependence of the Magnetic Susceptibility for a Spin Glass

**Fig. 2:**
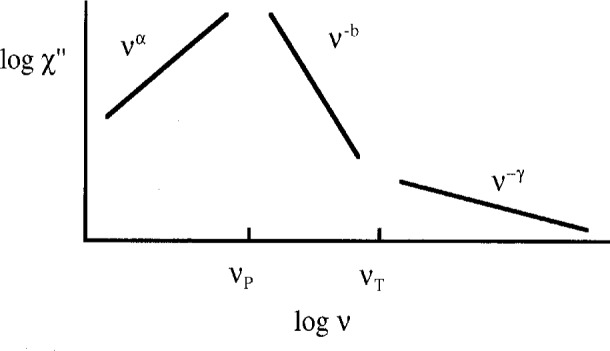
A schematic diagram showing the three distinct frequency regimes for the imaginary part of the magnetic susceptibility of a paramagnetic susceptibility of a paramagnet above the spin-glass transition.

In a study, of the dipolar-coupled, Ising spin glass, LiHo_0.167_Y_0.833_F_4_, the shape of the magnetic susceptibility, *x* (*v*) = *x*′(*v*) + *ix*″(*v*), was found to be qualitatively similar to that of the dielectric susceptibility of supercooled liquids discussed above. As was the case for the liquids, the imaginary part of the susceptibility, *x*″(*v*), is characterized by three distinct regimes as shown in Fig. 2: one power law in frequency, with positive exponent *α*, below the peak at *v*_P_; another power law, with negative exponent −*b*, just above *v*_P_; and, finally, a third power law with exponent −*γ* in the high-frequency limit. Measurements demonstrate that the exponent of the high-frequency power law goes to zero as *T* → *T*_sg_(*H*_t_). The transition temperature was determined *directly* from the divergence of the nonlinear susceptibility *x*_3_′ [14]. Here *H*_t_ is a transverse field which can be used to tune the characteristic response times of the system (as well as the spin-glass transition temperature itself). Thus, the high-frequency behavior is very similar to what we described above for glasses.

For the spin glass it is unnecessary to extrapolate the results from a much higher temperature down to the spin-glass transition since the data can be taken directly at temperatures very close to *T*_sg_. Consequently, the fact that the exponent for the high-frequency power law is zero at the transition temperature, is not dependent on any extrapolation procedure. By applying a transverse magnetic field, it is possible to shift the spin-glass transition temperature. When this is done, the temperature at which the high-frequency response becomes frequency independent shifts to the new value of *T*_sg_. We argue that since this frequency-independent signature is seen in a spin glass at the transition, we should associate a constant high-frequency tail in liquids with an underlying phase transition in that system as well.

Not only does the high-frequency behavior become frequency independent as the spin-glass transition is approached, but, as distinct from liquids, the exponent, *α*, of the power law below *v*_P_ also approaches zero. A frequency independent spectrum in *x*″ as *v* approaches zero corresponds to 1/*f* = frequency noise in the magnetization by the fluctuation-dissipation theorem, and has been used to define the onset of the spin-glass state [15].

## 5. Divergence in the Linear Susceptibility of a Spin Glass

We can use the same arguments in calculating the static magnetic susceptibility of the spin glass as we used above to analyze the static dielectric susceptibility of the liquid. We again use the Kramers-Kronig relation:

$$x^{\prime }(0)=\frac{2}{\pi }\int_{-\infty }^{\infty }x^{\prime \prime }(\nu )\;d\;\ln\;\nu .$$ (5)

However, now we have *x*″(*v*) being frequency independent both below as well as above *v*_P_. This again gives rise to a divergence of the static susceptibility, an unexpected result for the linear, as opposed to the nonlinear, susceptibility. (The assumption here is, of course, that the low-frequency behavior is flat down to zero frequency.) Although this argument predicts a divergence in *x*, it is a very weak one as a function of frequency. From a flat spectrum in *x*″(*v*) we would expect

$$x^{\prime }(\nu \to 0)\propto -\log\;\nu .$$ (6)

Thus, the expectation is that *x*′(*v* → 0) would grow logarithmically slowly as the measuring frequency is decreased. Data that exist in the literature on the dependence of *x*′(0) on the measurement frequency do in fact show a small and monotonic rise in its value as the frequency is decreased. In all cases that we have investigated [16], with one exception [17], the value of the static susceptibility does in fact appear to vary essentially as predicted by Eq. (6). Since a logarithmic growth of the susceptibility with decreasing frequency is very hard to measure, it is perhaps not surprising that such an effect could be overlooked in the data. However, the above analysis of the imaginary part of the spectrum necessarily implies a divergence in the linear, as well as the nonlinear, susceptibility of spin glasses.

## 6. Conclusions

The available evidence from susceptibility measurements on both supercooled liquids approaching the glass and paramagnets approaching the spin glass suggests that some of the standard lore in this field may not be correct. In both systems, as well as in a plastic crystal [18], there is a tail in the susceptibility response extending out to high frequencies. Thus, this seems to be a robust feature of the spectrum in disordered systems. In both glasses and spin glasses we find a signature of freezing of the dynamics in this high-frequency response of the sample. Thus it is not only the very lowest frequencies but also the high frequencies that are important for studying the freezing transition. This is advantageous for computer simulations of these systems since high frequencies, corresponding to short times, are much more accessible than low ones in a simulation.

We have argued as well that supercooled liquids do have a diverging static susceptibility which would indicate that there is an underlying phase transition with a growing correlation length that mediates the transition into the glass. Although these conclusions are based on extrapolations of the data over a significant range of temperature, they are perhaps some of the most accurate data that presently exist in this field so that their extrapolations should be taken very seriously. We also note that, since similar behavior in the high-frequency susceptibility is found in an insulating spin glass, we have added reason to believe that a frequency-independent high-frequency tail in the susceptibility is an independent signature of a phase transition in a disordered system.

Finally we have argued that the data on the imaginary part of the magnetic susceptibility of a spin glass indicates that there is a divergence in the linear response of this system. The divergence would be very weak, logarithmic in frequency. Data taken from the literature appear to be consistent with this prediction.

## Footnotes

3 A more extensive version of this paper will be published in *Advances in Physics.*
